# Surface Properties and Architectures of Male Moth Trichoid Sensilla Investigated Using Atomic Force Microscopy

**DOI:** 10.3390/insects13050423

**Published:** 2022-04-30

**Authors:** Thomas Charles Baker, Qiong Zhou, Charles E. Linn, James Y. Baker, Timothy B. Tighe

**Affiliations:** 1 Department of Entomology, Huck Institute for Life Sciences, Penn State University, University Park, PA 16802, USA; semaj22@gmail.com; 2 College of Life Science, Hunan Normal University, 36 Lushan Road, Changsha 410081, China; zhoujoan2004@163.com; 3 Department of Entomology, Cornell AgriTech, Cornell University, Geneva, NY 14456, USA; cel1@cornell.edu; 4 Huck Institute for Materials Science, Penn State University, University Park, PA 16802, USA; tbt1@psu.edu

**Keywords:** moth sex pheromones, male moth antennae, olfactory sensilla, trichoid sensilla, atomic force microscopy, surface potential, insect epicuticle

## Abstract

**Simple Summary:**

The capture and transport of pheromone odorants to olfactory sensory neurons (OSNs) via the surfaces of thousands of antennal sensory hairs of male moths are the first and requisite steps that permit olfaction and upwind flight orientation to females to occur. Our investigations of the male pheromone-sensitive sensory hairs (trichoid sensilla) from five moth species using atomic force microscopy (AFM) revealed differences in the densities, heights and depths of pores and ridges involved in pheromone odorant capture and transport. Measurements of electrical surface potentials across sensilla in our study suggests that there is a heterogeneity in the distribution of surface lipids between ridges, pores and inter-ridge areas that likely facilitate the capture and transport of pheromone odorants to OSNs. Controlled heating of sensilla revealed that heating did not melt or change the shapes of small lipid exudates residing within the pores or of large exudates completely covering the pores. These results suggest that such exudates are crystalline wax blooms that comprise a different form of lipid than the free lipid monolayer that covers the rest of these olfactory hairs.

**Abstract:**

The surfaces of trichoid sensilla on male moth antennae have been sculpted over evolutionary time to capture pheromone odorant molecules emitted by the females of their species and transport the molecules in milliseconds into the binding protein milieu of the sensillum lumen. The capture of pheromone molecules likely has been optimized by the topographies and spacings of the numerous ridges and pores on these sensilla. A monolayer of free lipids in the outer epicuticle covers the sensillar surfaces and must also be involved in optimal pheromone odorant capture and transport. Using electro-conductive atomic force microscopy probes, we found that electrical surface potentials of the pores, ridges and flat planar areas between ridges varied in consistent ways, suggesting that there is a heterogeneity in the distribution of surface lipid mixtures amongst these structures that could help facilitate the capture and transport of pheromone molecules down through the pores. We also performed experiments using peak force atomic force microscopy in which we heated the sensilla to determine whether there is a temperature-related change of state of some of the surface lipid exudates such as the prominent domes covering many of the pores. We found that these exudates were unaffected by heating and did not melt or change shape significantly under high heat. Additionally, we measured and compared the topographies of the trichoid sensilla of five species of moths, including the distributions, spacings, heights and diameters of ridges, pores and pore exudates.

## 1. Introduction

For tens of millions of years male moth sex pheromone olfactory systems have been under evolutionary pressure to become optimally designed to acquire and discriminate relative abundances of female-emitted sex pheromone molecules in species-specific blends [1]. In order for the olfactory process to begin, the surfaces of each of the trichoid sensilla housing olfactory sensory neurons (OSNs) must capture these molecules and facilitate their transport to the binding proteins and OSNs in the sensillum lumen [2]. A monolayer of free lipids [2,3,4,5] covers all surfaces of the insect epicuticle and comprises the middle layer of the outer epicuticle. This free lipid monolayer in the epicuticle has been implicated as being the main barrier preventing an insect from desiccating. On antennal sensilla such as trichoid sensilla, however, the epicuticle must also be sufficiently permeable to lipophilic odorant molecules so that these can be efficiently captured and transported into the interiors of sensilla [3].

For the past 50 years or so there have been sporadic but intense efforts investigating the properties of the trichoid sensilla epicuticle with its pores, pore tubules and ridges to understand how pheromone molecules are transported to interact with binding proteins in the sensillar lymph [6,7,8,9,10,11,12,13,14,15,16,17]. Our previous work [16], employing atomic force microscopy (AFM), suggested that there is a heterogeneity of lipid coatings that provides a gradient from the sensillar ridges to the pores that could aid in such capture and transport. A companion study [18] showed that extracts of male corn earworm (*Helicoverpa zea*) antennae, imbued with thousands more trichoid sensilla than females, have a set of more polar, branched hydrocarbons than do extracts of female antennae. This finding suggested that *H. zea* male trichoids are specially coated with a class of lipids that could selectively optimize the capture of their female-emitted aldehyde pheromone components. Such a mechanism would perform as a type of “olfactory lens” as suggested by Kaissling [10] for the domestic silk moth (*Bombyx mori*), focusing the sex pheromone molecules of this species preferentially onto trichoid sensilla.

In this study, we investigated with AFM techniques the pore-ridge-structured surfaces of trichoid sensilla on males of five species of moths. In some of the recordings we used frequency modulated Kelvin probe force microscopy (FM-KPFM) to measure the surface potential differences across sensillar ridges, pores and the dome structures that we observed completely covering some of the pores. We also performed heating experiments to determine whether the surface lipids would change their shapes upon changes in temperature. We wanted to understand some of the charge distribution properties and possible lipid phase changes of these structures that might contribute to pheromone molecule capture and transport.

Previous AFM studies by Maitani et al. [16] for *H. zea* and that of Su et al. [17] for *B. mori* showed that the pores and ridges of trichoids are intimately associated. The topographies of ridges and pores were implicated as being designed to optimize aerodynamic trapping of pheromone [17] and also for forming lipid ridge–pore gradients to move pheromone odorants to the pores [16]. Thus, ridges and pores have been implicated as working together for the optimal subsecond reporting of pheromone component blends in the strands of point-source pheromone plumes [19].

## 2. Materials and Methods

### 2.1. Obtaining Cut Sensilla from Male Moths

#### Moths

Three of the five species used in this study—*H. zea*, the tobacco hawk moth (*Manduca sexta*) and the spongy moth (*Lymantria dispar*)—were obtained as pupae from laboratory-grown moths at Penn State University, separated by sex, and allowed to emerge as male adults in separate 30 cm × 30 cm screen cages. Males of the two strains of the European corn borer, *Ostrinia nubilalis*, were obtained as pupae from Cornell AgriTech. Pupae of *O. nubilalis* were separated by sex, flown via courier express to Penn State and allowed to emerge as male adults in the above-described screen cages. Males of the winter moth, *Operophtera brumata*, were obtained as adults from the Department of Entomology at the University of Massachusetts. The males had been captured during the previous winter in sex pheromone traps baited with the pheromone of this species, removed from the traps and stored in plastic bags in a freezer until shipment to Penn State where the bags were kept in a freezer at −20 °C until use.

### 2.2. Piezo Device for Shaving the Sensilla from Intact Sensilla

The antennae of male *H. zea*, *M. sexta*, *O. nubilalis* and *L. dispar* were removed with scissors from cold-immobilized living moths. Trichoid sensilla were then shaved from the antennae using a piezo device with a piezo crystal vibrating a glass capillary fashioned after the device of Hillier et al. [20]. Because we wished to use AFM to examine the natural epicuticular lipid surfaces of sensilla with their lipid coatings and exudates intact, the cut sensilla were not treated (preprocessed) with solvents or in any other fashion. The shaved sensilla were allowed to land on sticky pads (see below) and were examined by AFM with no further manipulations. Trichoid sensilla of male *O. brumata* were excised after the frozen males had been removed from the freezer in their bags and acclimated to room temperature for more than 30 min. The glass capillary-knife was driven by a function generator to vibrate at variable high frequencies and amplitudes that were adjusted to successfully cut the trichoid sensilla of the different species having different toughness and elastic properties. Under a Leitz stereo microscope, the vibrating tip of the glass knife was brought into contact with the sensilla at as close to their bases as could be discerned through the microscope. We were not able to shave the sensilla precisely at their bases, but usually we were able to cut them at a sufficiently low point on such that more proximal basal features could be compared with those more toward the tip.

The sensilla were excised such that they would land on double-sided sticky pads (STKYDOT, Bruker AFM Probes, Camarillo, CA, USA) and were mounted to 15 mm stainless steel disks (Ted Pella, Inc., Redding, CA, USA). For FM-KPFM studies measuring the surface potential of sensillar domes, pores and ridges, the sensilla were shaved to land on 12 mm carbon conductive tabs (PELCO Tabs, Ted Pella, Inc., Redding, CA, USA).

### 2.3. Bruker Peak-Force AFM Scanning Process

In all our studies we used a Bruker Dimension Icon AFM instrument (Bruker Nano Inc., Santa Barbara, CA, USA). The topography scans were collected in PeakForce Tapping mode using a ScanAsyst Air probe (0.4 N/m spring constant, Bruker AFM Probes, Camarillo, CA, USA) with a 0.5–2 nN setpoint. Care was taken to align the scans parallel to the long axis of the sensilla to avoid contaminating the probe with adhesive from the double-sided sticky tape. The surface potential scans were done in PeakForce Kelvin Probe Force Microscopy frequency modulated mode (PF-KPFM-FM) using a PFQNE-AL probe (0.8 N/m spring constant, Bruker AFM Probes, Camarillo, CA, USA) with a 0.5–2 nN setpoint and a 25 nm lift height. The same care was taken to align the scans parallel to the long axis of the sensilla to avoid contaminating the probe with adhesive from the double-sided carbon conductive tabs. All data analysis was performed in Nanoscope Analysis 2.0 (Bruker Nano Inc., Santa Barbara, CA, USA).

### 2.4. Methodology for Locating and Engaging the Tip onto Single Sensilla

For each trial for each species on a given day, discs potentially containing shaved sensilla were first observed under a standard binocular microscope to determine before AFM scanning whether there were sufficient numbers of sensilla on the disc amidst the large numbers of antennal cuticular scales that always dropped onto the discs during piezo-shaving. The process was then the same for all of the species: a good disc containing a large number of easily located trichoid sensilla would be placed within the AFM chamber and the door closed. The AFM probe-tip would be positioned to land on a section of a sensillum and a low-resolution preview image would be obtained to determine the quality of the scanned area with regard to numbers of pores, domes and ridges for later analysis. Inappropriate areas were ignored due to either contamination/debris on a sensillum or the instability of the sensillum due to how it landed on the tape during sample prep, and new low-resolution scans of new areas were performed until a good location was found. Good prospective sections were imaged in higher resolution (512 samples/line, setpoint and tip velocity optimized).

### 2.5. Surface Potential Scans

PeakForce KPFM was performed exactly as described above and a low resolution topography scan of a prospective area was performed to assess its quality for the pores, domes and ridges we were interested in. Once a good area was found, a high-resolution scan was carried out that provided both topography information as well as surface potential readings of the area.

### 2.6. Heating Experiment Scans

For experiments examining the effects of heating on sensilla, scans were first carried out at 23 °C (room temperature). The AFM tip was brought into contact with a section of a sensillum, as described above, and a low resolution topography scan of a prospective area was carried out to assess its quality regarding the pores, domes and ridges we were interested in. Once a good area was found, a high-resolution scan was performed. The AFM tip was withdrawn, and the chamber was programmed to increase its temperature to 50 °C. When this temperature was reached, the AFM tip was descended and brought into contact with the sensillum again, and several low-resolution scans were performed to attempt to locate the same section of the sensillum with the same array of pores and domes that was scanned earlier at room temperature. When this area was located, a high resolution scan was performed at 50 °C.

Over the course of this study, we took 171 AFM image-scans, with 63 different male moth trichoid sensilla being used for our analyses.

### 2.7. Measurements

During our measurements of the trichoid sensilla of the five moth species, we encountered a variety of pores that appeared completely open, some having small domes (“nipples”) lying within the pore and beneath its surface and pores that were fully covered by a large dome. We placed these into three categories: “open pores”, “nipple-pores”, and “domed pores”.

**Figure 1:**
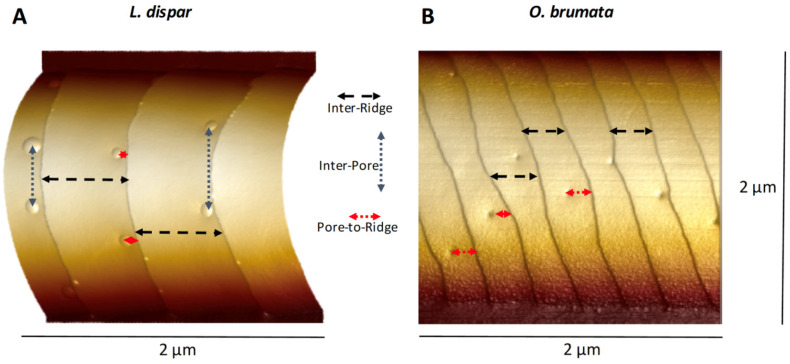
AFM 3D images of portions of *Lymantria dispar* and *Operophtera brumata* trichoid sensilla illustrating how pore and ridge distributions and distances were measured. (**A**) *L. dispar* here exhibits only domed pores in this section. Inter-pore distance-measuring criteria are indicated by dashed black vertical arrows, inter-ridge distance-measuring criteria by dashed black horizontal arrows and pore-to-ridge distance-measuring criteria by red dashed horizontal arrows. (**B**) *O. brumata* here exhibits only open pores. There is only one pore per inter-ridge flat planar area visible in this section and so no inter-pore distances could be measured. Criteria for measuring inter-ridge and pore-to-ridge distances are same as for *L. dispar*.

**Figure 2:**
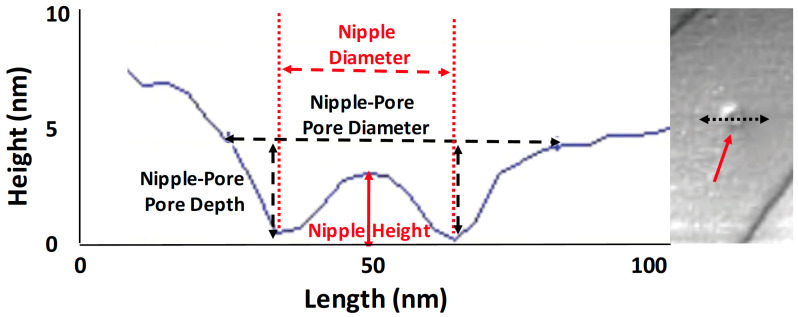
An AFM section taken through an *Operophtera brumata* nipple-pore using the Nanoscope Analysis “section” tool, illustrating how measurements were taken of pore diameter and depth, nipple diameter and height. Inset on the right shows the nipple pore (red arrow) that was measured, and the black double arrow shows the orientation of the section.

**Figure 3:**
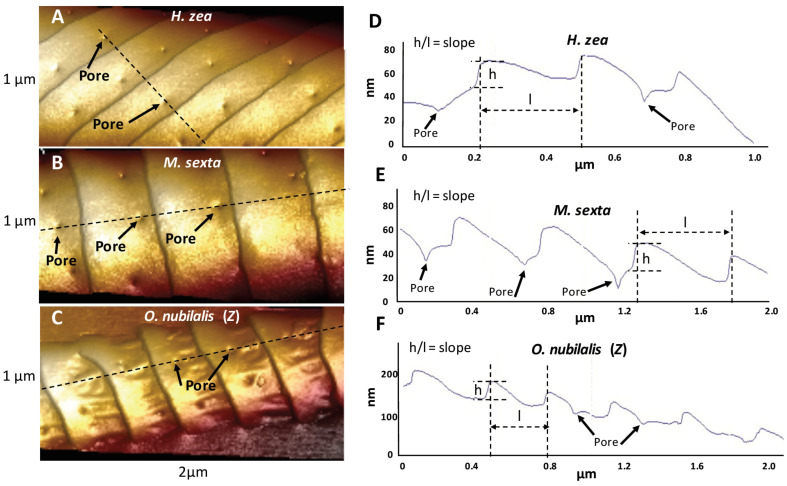
Illustration of how ridge heights were measured using the Nanoscope Analysis section tool. (**A**–**C**) AFM 3D images of portions of trichoid sensilla of *Helicoverpa zea*, *Manduca sexta* and *Ostrinia nubilalis Z*-strain. Open pores are indicated by black solid arrows. The paths in linear sections that were obtained across different AFM scans using the section tool are shown with dashed black lines. Note the crossing of numerous pores and ridges by the section tool. (**D**–**F**) Corresponding cross-sectional line drawings produced by the section tool for *H. zea*, *M. sexta* and *O. nubilalis*, respectively. The “h” noted in each figure shows the features that the Nanoscope Analysis section tool used to produce digital readouts of ridge heights, and the “l” illustrates how the section tool provided inter-ridge distance readouts. The locations of the same pores seen in panels (**A**–**C**) that were intersected by the section tool (black dashed lines) are indicated with black arrows in panels (**D**–**F**).

All of the trichoid sensilla from all species exhibited abundant ridges that varied in spacing, height and slopes of the flat planar areas between successive ridges. The regimes for measuring the distances between successive ridges (“inter-ridge distance”), the distances between pores (“inter-pore distance”) and distances between a pore and the nearest steep ridge-cliff (“pore-to-ridge distance”) are depicted in Figure 1. The Nanoscope Analysis line-measuring tool was used to measure these distances in 2D plan view (flat) mode. Pore density was measured using the Nanoscope Analysis “step” tool to demarcate individual flat inter-ridge areas containing pores. The surface areas varied slightly in each flat area demarcated by the cursor for analysis, but the amount of surface area was then easily calculated using the step tool and then was adjusted to a standard “pores/µm^2^”. We used the “section” tool from the Nanoscope Analysis software package to measure nipple-pore pore diameters and pore depths, nipple-pore nipple heights and diameters (Figure 2), open pore depths and diameters, and domed pore dome heights and diameters. Ridge heights and inter-ridge slopes were also measured using the Nanoscope Analysis section tool (Figure 3). When the Nanoscope Analysis section tool was used, two cursors were aligned so that the software provided precise digital nanometer readouts of the vertical height of each ridge, nipple or pore as well as the horizontal distances between ridges, or pore and nipple edges.

## 3. Results

### 3.1. Ridges and Pores Overview

**Figure 4:**
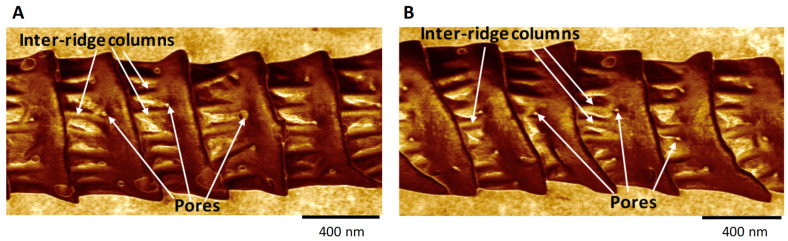
AFM adhesion mode 2D images of the trichoid sensilla from *Ostrinia nubilalis E*-strain (**A**) and *Z*-strain (**B**), highlighting the unusual inter-ridge columns typical of this species but missing in all the other species used in this study. A few of the many inter-ridge columns are indicated by white arrows from the top, and locations of some pores are denoted by white arrows from the bottom.

We performed AFM topographical scans of the trichoid sensilla of each species to determine whether there were any obvious species differences that might be related to the type of major pheromone component used by each species. Our results (see later in this section) showed that sensilla varied with regard to the spacing of ridges along their lengths and also with the spacings and densities of the pores along the flat planar areas of the surfaces between the ridges (Figure 1 and Figure 3). There appeared to be species differences in the distances of pores from the base of each ridge’s steep “cliff” that drops down onto the next-most-distal planar area toward the tip (Figure 3). Images of the trichoid sensilla of all species exhibited similar morphologies of fairly smooth inter-ridge surfaces except for both *O. nubilalis E* and *Z* strains. For *O. nubilalis* there were peculiar inter-ridge columns, projecting approximately perpendicular to the ridge-cliffs, which seemed to provide extra troughs in which the pores resided (Figure 3C and Figure 4). The sensilla of the *E* and *Z* strains of *O. nubilalis* were examined separately because their major pheromone components are the *E* and *Z* geometrical isomers, respectively, of 11-tetradecenyl acetate and as such have different 3D shapes that could possibly interact differently with particular sensillar architectures.

**Figure 5:**
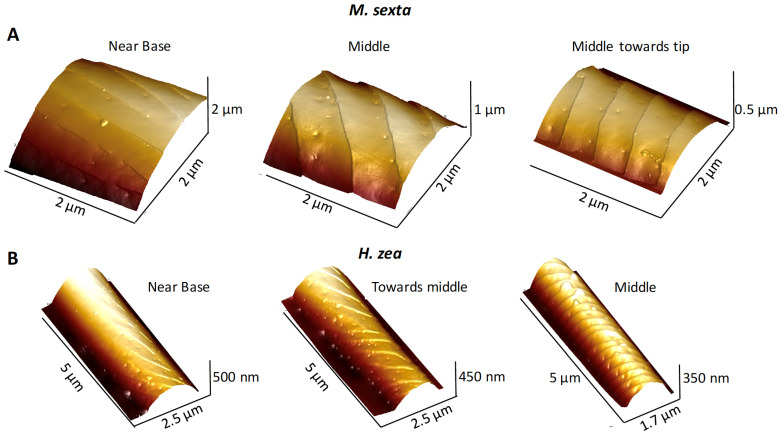
Illustration of how sensillar ridges are oriented more longitudinally with respect to the longitudinal axis of a sensillum near its base to become oriented more transversely more distally toward the tip of the sensillum. (**A**) Three AFM 3D images of a single *Manduca sexta* trichoid sensillum taken successively from near its base (left) to an area more distally toward its tip. (**B**) Three AFM 3D images of a single *Helicoverpa zea* trichoid sensillum taken successively from near its base to an area more distally toward its tip.

Our images showed that pores and ridges are intimately associated (Figure 5), a finding previously reported [16,17] in series of images taken more proximally toward the bases of the sensilla compared to more distally toward the tips. Near the base, ridges were nearly absent and there were few pores (Figure 5).

Atomic force microscopy images of nipple-pores detected a small exudate within the pore, i.e., the “nipple”, lying within an otherwise open pore, whose dimensions we measured using the section tool (Figure 2). For the open pore classification, the AFM detected no such internal exudate. For domed pores, the exudate was so large that it encompassed the entire diameter of the pore, and no external boundaries of the pore’s rim could be detected (Figure 1A). In our samples, the relative abundances of these three types varied across species as well as within samples of the same species.

### 3.2. Surface Potential of Domes, Pores and Ridges

**Figure 6:**
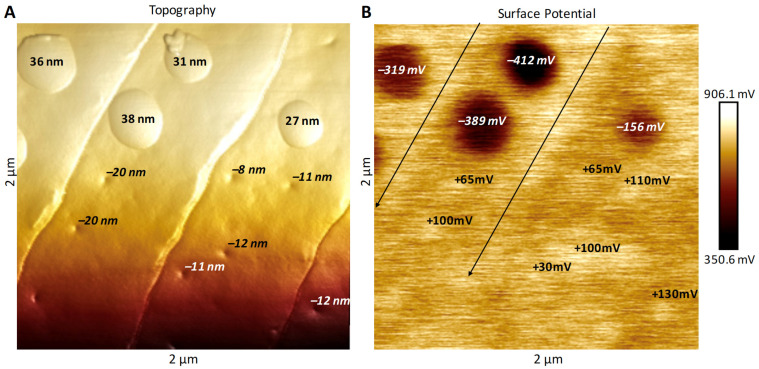
Surface potential measurements of a 2 µm × 2 µm section of a trichoid sensillum from *Manduca sexta* related to the topography of ridges, domes and pores on the same section. (**A**) AFM 3D topographical image showing heights of domes of domed pores and depths of open pores. Height/depth measurements were taken using the Nanoscope Analysis section tool. (**B**) Surface potentials of the same domed-pore domes and open pores shown in (**A**). Surface potential measurements were taken using the Nanoscope Analysis section tool. Note the domes have large negative potential readings relative to the background potentials of the neighboring flat planar regions, whereas open pores exhibit small positive potentials. The long black parallel arrows in (**B**) delineate small positive potentials running along the ridges.

**Figure 7:**
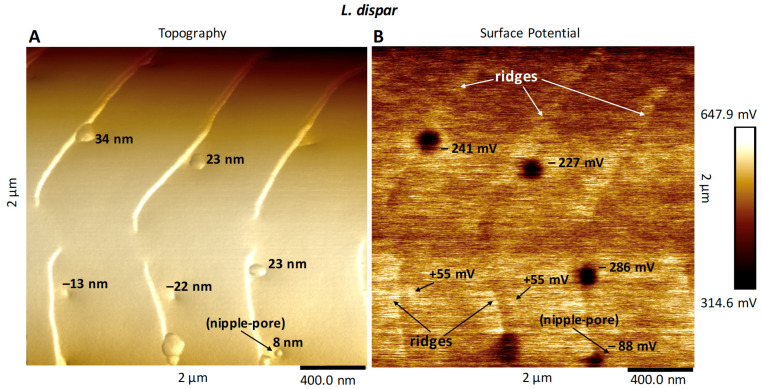
AFM surface potential readings from a 2 µm × 2 µm section of a *Lymantria dispar* trichoid sensillum related to height of domes, depth of open pores, and height of ridges. Note the mesh area of ridges in these images, incompletely crossing the surface of the sensillum. (**A**) Topography scan of the sensillum. Heights of domes and depths of pores are indicated on the figure. (**B**) Corresponding surface potential scan of this same section. Note the negative surface potentials of domes and positive surface potentials of pores. The ridges stand out as having more positive surface potentials than the flat planar inter-ridge regions.

**Figure 8:**
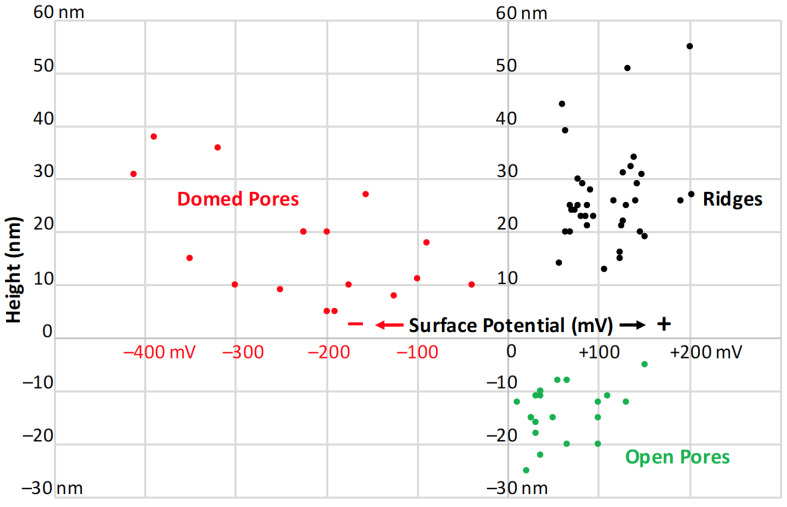
Scatter diagram of individual surface potential readings from the domed pore domes, the open pores and the ridges on the sensilla of *Helicoverpa zea*, *Manduca sexta*, *Lymantria dispar* and *Operophtera brumata*. For ridges, three, two, two, and one sensilla were used with 10, 9, 5, and 12 surface potential measurements taken, respectively. For open pores, one, two, one, and one sensilla were used with 2, 12, 3, and 2 surface potential measurements taken, respectively. For domed pores, 8, 4, 3, and 1 surface potential measurements were taken, respectively.

Our goal for these measurements was to use electrical surface potential readings to determine the degree of uniformity of the surface lipid layers across these species’ sensilla. These measurements would serve as a follow-up to the conclusions of Maitani et al. [16], demonstrating that some species’ sensilla did exhibit lipophilic/hydrophilic heterogeneity across their surfaces. Our results showed that the full domes of *M. sexta* (Figure 6), *L. dispar* (Figure 7), *H. zea* and *O. brumata* all exhibited negative surface potentials relative to the mean surface potentials of the neighboring flat planar areas. The size of negative surface potential appeared directly related to the heights of the domes (Figure 8). The mean (background) surface potentials of the flat inter-ridge areas of *M. sexta*, *L. dispar* and *O. brumata* were all positive: 541 mV (±43.4 S.D.; N = 15), 595 mV (±56.4 S.D.; N = 8) and 572 mV (±23.4 S.D.; N = 8), respectively. The mean surface potential of the *H. zea* flat planar regions (444 mV (±37.4 S.D.; N = 13) was lower than the levels from the other species.

The open pores of these four species exhibited a positive surface potential relative to the neighboring sensillar regions (Figure 8). The level of positive potential appeared to be greater with greater pore depths. The ridges also exhibited a positive surface potential compared to the neighboring planar areas (Figure 6, Figure 7 and Figure 8).

We could not measure the surface potentials of the nipple-pore nipples—they resided wholly within each pore below the pore’s rim—but some of the smaller-sized domes did also exhibit negative surface potentials (Figure 8). That the surface potentials of dome-exudates varied directly with size could mean that the very small exudates comprising nipple-pore nipples have the same chemical composition as domes.

### 3.3. Effect of Heating on Domes and Pores

**Figure 9:**
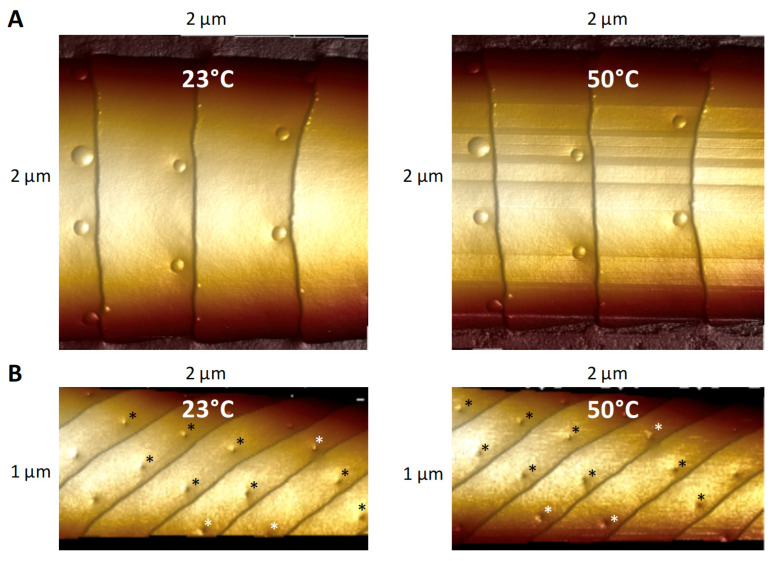
AFM 3D images taken from the heating experiment. (**A**) Image of a 2 µm × 2 µm portion of a *Lymantria dispar* sensillum showing domed pores at 23 °C and then after heating to 50 °C. No apparent changes in the domed pores are visible after heating. (**B**) Image of a 1 µm × 2 µm portion of a *Helicoverpa zea* sensillum showing open pores at 23 °C and then after heating to 50 °C. Pores that are labelled with black or white asterisks in the 50 °C image are the same pores appearing with black or white asterisks in the 23 °C image. No apparent changes in the open pores occurred after heating.

**Figure 10:**
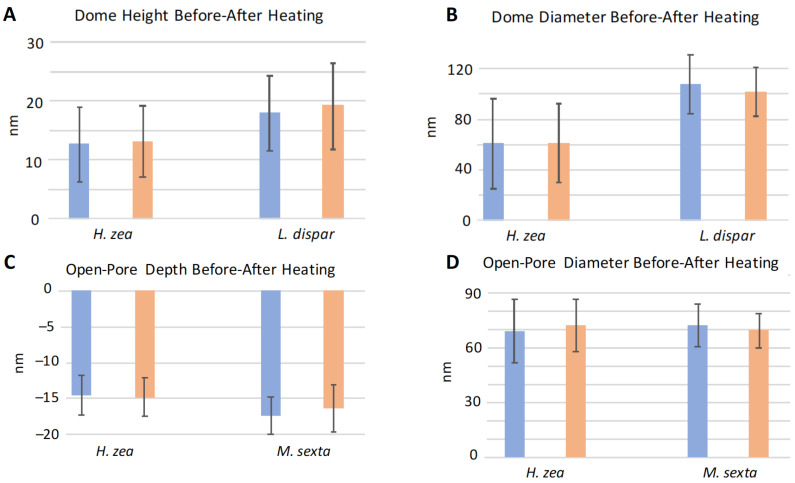
Effect of heating on mean heights, depths and diameters of domed-pore domes and open pores of *Helicoverpa zea*, *Lymantria dispar* and *Manduca sexta* trichoid sensilla. Blue histogram bars indicate measurements taken on sensilla at 23 °C. Orange bars indicate measurements taken on the same sensilla after heating to 50 °C. (**A**,**B**) N = 18 domes measured for *H. zea* and N = 10 for *L. dispar*. (**C**,**D**) N = 11 open pores measured for *H. zea* and N = 10 for *M. sexta*. Error bars denote standard deviation.

**Figure 11:**
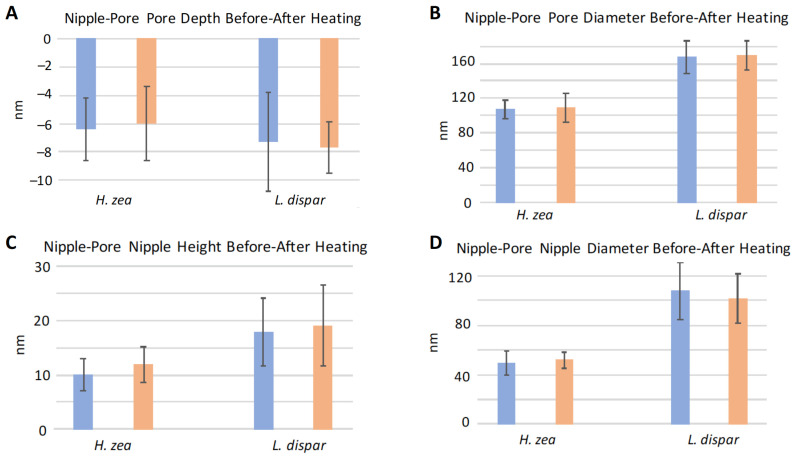
Effect of heating on mean heights, depths and diameters of nipple-pore pores and nipple-pore nipples on *Helicoverpa zea* and *Lymantria dispar* trichoid sensilla. Blue histogram bars indicate measurements taken on sensilla at 23 °C. Orange bars indicate measurements taken on the same sensilla after heating to 50 °C. (**A**,**B**) N = 23 nipple-pore pores measured for *H. zea* and N = 10 for *L. dispar*. (**C**,**D**) N = 23 nipple-pore nipples measured for *H. zea* and N = 10 for *L. dispar*. Error bars denote standard deviation.

We performed experiments in which we heated sensilla of different species to see whether structures such as the domes covering pores would melt or change shape at higher temperatures related to flight-muscle-related warming of the thorax and antennae [21,22]. This experimental increase in temperature was designed to slightly exceed even the thoracic temperatures (nearly 40 °C) known to be produced by flight muscle activity in pheromone-stimulated male moths [21,22]. We found that heating of the sensilla from room temperature (23 °C) to 50 °C did not cause any apparent changes in the heights or diameters of domes (Figure 9A and Figure 10A,B). The 50 °C temperature also did not result in enlargement of the depths or diameters of open pores or to cause interior substances to exude from the pores (Figure 9B and Figure 10C,D). The dimensions of the nipple-pore nipples and pores remained similarly unaffected by this increase in heat (Figure 11). The lack of heating-related change in nipple-pore nipples suggests that the composition of nipple exudate is similar to that of domes.

### 3.4. Pore and Ridge Heights, Spacings, and Densities

#### 3.4.1. Pore Densities, Dimensions and Proximity to Ridges

**Figure 12:**
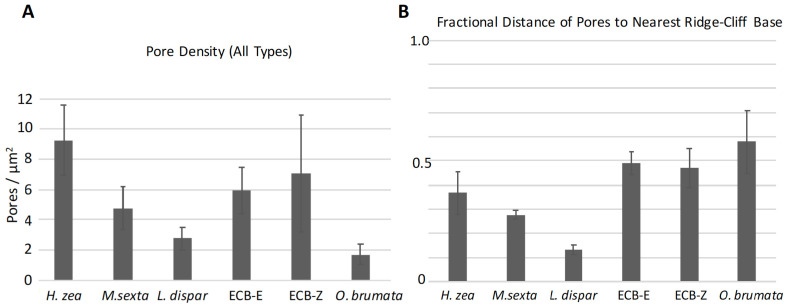
Mean pore densities (**A**) and the mean fractional distances of pores from nearest proximal ridge-cliffs (**B**) on the trichoid sensilla of five moth species. All measurements were performed on the middle regions of the sensilla. The labels, “ECB-E” and “ECB-Z”, refer to the *E*- and *Z*-strains, respectively, of *Ostrinia nubilalis*. Error bars denote standard deviations. (**A**) N = 11, 8, 12, 10, 10, and 10 densities for *Helicoverpa zea*, *Manduca sexta*, *Lymantria dispar*, *O. nubilalis E*-strain, *O. nubilalis Z*-strain, and *Operophtera brumata*, respectively. (**B**) N = 14, 19, 16, 7, 10, 10, and 10 for these same species.

We were interested in using AFM topographical information to determine whether we could find any characteristic pheromone-related species differences in the height-architectures and spacings of ridges and pores on trichoid sensilla. We measured the distances of pores from each other arranged on each inter-ridge planar surface (Figure 1) and also calculated the pore densities on each surface regardless of pore type. Two species, *L. dispar* and *M. sexta,* exhibited the lowest pore densities of all species except for *O. brumata* (Figure 12A). In *O. brumata*, there was usually only one pore per planar area and so no inter-pore distances could be measured. The pores of all types in all species, except for those of *L. dispar*, were similarly spaced from each other at ca. 350–400 nm (Figure S1A). Those of *L. dispar* were spaced 602 nm from each other (±104.3 S.D.; N = 17) (Figure S1A).

On a fractional basis the pores of *L. dispar* were only ca. 12% of the way out onto the flat inter-ridge planar areas from the base of the nearest proximal ridge-cliff (Figure 12B). The pores of *M. sexta* and *H. zea* were 28% and 37% of the way out onto the flat planar areas, respectively (Figure 12B). On the other hand, the pores of *O. brumata* and those of the *E*- and *Z*-strains of *O. nubilalis* were positioned in such a way that they were placed farther out on the flat planar areas approximately mid-way between successive ridges (Figure 12B). The measured linear distances of the pores of these three species from their nearest respective ridge-cliff bases (Figure 1) were between 155–179 nm (Figure S1B). The pores of *H. zea* and *L. dispar* were closer in linear distance to the bases of the nearest proximal ridges (103 nm ± 35.5 S.D. and 74 nm ± 19.4 S.D.; N = 14 and 16, respectively) than those of the other species (Figure S1B).

Among both strains of *O. nubilalis* and *O. brumata* the diameters (Figure 2) of nipple-pore pores (73, 74, 69 nm, respectively) and open pores (63, 63, 66 nm, respectively) were of similar size and smaller than those of *M. sexta* (110 nm nipple pore, 97 nm open pore) and *L. dispar* (164 nm nipple pore, 75 nm open pore) (Figure S2A,B). Not surprisingly, the pore depths (Figure 2) of nipple-pore pores (Figure S3A) were not as great as those of open pores (Figure S3B) due to the existence of nipple-pore exudates (nipples) at the bottom of the nipple-pores (Figure 2). The heights and diameters of nipples (Figure 2) in nipple-pores were, as expected, smaller than the heights and diameters of domes (Figures S4 and S5) due to the lesser amount of pore exudate in nipple-pores compared to domed pores.

#### 3.4.2. Ridges

Although ridges appear to possibly be spiraling up toward the tip, they do not comprise a continuous spiral, but rather have a mesh-point where they stop (Figure 7), as was previously observed by Su et al. [17] for the sensillar ridges of *B. mori.* Along the regularly occurring ridges completely spanning a sensillum transversely relative to its long axis—the only type of ridge arrangement that we used for our measurements—the ridges of all species exhibited recurring, steep, distally oriented height-drops (“ridge-cliffs”) (Figure 3D–F). Along these stepwise ridge-cliff descents, the slopes of each flat area between the cliffs ascended and then abruptly dropped down to the next successive distal planar region (Figure 3D–F). The ridge structures and inter-ridge planar areas of both strains of *O. nubilalis* were different in appearance from those of the other species (Figure 3C and Figure 4). Not only were there peculiar inter-ridge raised columns running nearly perpendicular to the edges of the ridge-cliffs, the spaces between columns variously harbored pores (Figure 3C and Figure 4). In addition, the ridges exhibited enlarged smooth collars extending toward their ridge-cliffs (Figure 3C and Figure 4).

**Figure 13:**
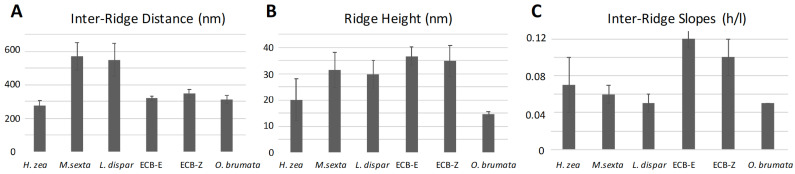
Mean inter-ridge distances, ridge heights and inter-ridge slopes of sensilla of five moth species. All measurements were performed on the middle regions of the sensilla. The labels, “ECB-E” and “ECB-Z”, refer to the *E*- and *Z*-strains, respectively, of *Ostrinia nubilalis*. Error bars denote standard deviations. (**A**) N = 14, 10, 16, 7, 10, and 10 measurements for *Helicoverpa zea*, *Manduca sexta*, *Lymantria dispar*, *O. nubilalis E*-strain, *O. nubilalis Z*-strain, and *Operophtera brumata*, respectively. (**B**) N = 13, 10, 10, 7, 10, 10 measurements for these same species. (**C**) N = 13, 10, 10, 7, 10, 10 measurements for these same species. Ratio “h/l” is height (h) of ridge-cliff divided by inter-ridge length (l).

The sensilla of *M. sexta* and *L. dispar* exhibited the greatest distances (569 nm and 550 nm, respectively) between successive ridges (Figure 13A). The inter-ridge distances of *H. zea*, *O. brumata* and the *E*- and *Z*- strains of *O. nubilalis* were all smaller and fairly similar to each other (275 nm, 313 nm, 319 nm, and 349 nm, respectively). The *E* and *Z* strains of *O. nubilalis* exhibited the greatest ridge-cliff height (37 nm and 35 nm, respectively) (Figure 13B), followed by those of *M. sexta* and *L. dispar* (32 nm and 30 nm, respectively). The inter-ridge planar surfaces of the two strains of *O. nubilalis* exhibited the steepest slopes (12% and 10% height/length ascent, respectively) toward the next-most-distal cliff-ridges than any of the other species, which all exhibited a height/length ascent of less than 7% (Figure 13C).

## 4. Discussion

The epicuticle of trichoid sensilla, just as is found over the entire surface of the insect body, is infused with a monolayer of free lipid that has issued from the sensillar lumen and diffused out into the epicuticle via pore tubules and pores [2,3,4,5]. These polar and nonpolar lipid mixtures may take many physical forms, including polar “water-lipid liquid crystals” and “crystalline wax surface blooms” [3]. The epicuticular free-lipid monolayer over the entire insect body stems from the outflow of lipids through pore channels [3,4] that have also been called “wax canals” [3]. In the wax canals of the insect body and by extension in the pores of insect olfactory sensilla the free lipids of the outer epicuticular layer are thought to be able to behave like lipid-water liquid crystals and change state to allow the movement of polar and nonpolar lipids outward and odorants inward, and then in an altered state to allow water to enter or exit the body [3].

On the trichoid sensilla of insect antennae, pores infused with this dynamic layer of free lipids are not hollow tubes through which adsorbed pheromone molecules surface-diffuse down the pore walls into the lumen; rather, the free lipids occupy the entire volume of the pore tubes and thus must facilitate the entry and transport of pheromone molecules into the sensillum lumen. Sex pheromone odorants, such as those of the five species used in this study, are all polar molecules with a long lipid tail and a polar functional group—or no functional group but double bonds—at the other end. The major (most abundant) pheromone components of the sex pheromones of these five species are: *H. zea*, (*Z*)-11-hexadecenal [23]; *M. sexta*, (*E,E*)-12,14-hexadecenal [24]; *L. dispar,* 2-methyl-7*R*,8*S*-epoxy-octadecane [25]; *O. nubilalis* “*E* strain”, 99% (*E*)-11-tetracecenyl acetate [26]; *O. nubilalis* “*Z* strain”, 97% (*Z*)-11-tetradecenyl acetate [27]; *O. brumata*, (*Z,Z,Z*)-1,3,6,9-nonadecatriene [28]. These polar molecules have both lipophilic and hydrophilic characteristics and thus should have the ability to behave as surfactants and solubilize into the epicuticular free lipid monolayer. Solubilized pheromone molecules could thus move down through the pores via a polarity-driven lipid gradient into the sensillum lumen.

Our PF-KPFM results indicate that there is heterogeneity in the local electric surface potential along different surfaces of the sensilla. FM-KPFM has been used to map the lateral surface potential differences in thin organic films [29,30]. In our study the domes over the pores exhibited a negative surface potential, usually in the range of hundreds of mV, relative to the neighboring epicuticular regions. The open pores, which have little to no obvious exudate compared to the pores covered with domes, exhibited a slightly higher local electric surface potential than neighboring regions. Additionally, the ridges of sensilla displayed a positive level of surface potential, similar to that of the open pores. Local electric surface potential is a result of the chemistry, i.e., the molecular structure of a surface, which plays a key role in its function and impacts how different types of charged molecules interact with the surface [29,30]. The heterogeneity in the surface potentials of trichoid sensilla and thus in the chemistries of the surface films comprising the epicuticular free-lipid layer impacts how polar sex pheromone molecules interact with the lipids in the epicuticular sensillar surface.

These preliminary KPFM results indicate that more work is needed concerning the chemical composition and formation of the lipids comprising the epicuticular surface films and pores of trichoid sensilla. These sensilla may be imbued with species-specific sets of epicuticular free lipids that can optimize the capture and transport of sex pheromone molecules peculiar to a species. Böröczky et al. [18] found that extracts of of *H. zea* and *Heliothis virescens* male antennae contained polar lipids such as esters of short-chain acids and long-chain secondary alcohols (C25–C32) that were not found in female antennal extracts of those same species. Kanaujia and Kaissling [9] found that sex pheromone molecule adsorption on the male antennae of the silk moth, *Antheraea polyphemus*, occurred preferentially in trichoid sensilla to produce a type of “olfactory lens” [10] that focused this special type of odorant onto the trichoids and not onto other parts of the antenna.

Our PF-KPFM data support the AFM phase-shift mode results of Maitani et al. [16]; that there is a heterogeneity of lipid coatings on the trichoid sensilla of some species of moths. That study [16] showed that there are differences in the lipophilicity and hydrophilicity of pores, domes and ridges of male *H. zea* trichoid sensilla. Locke [3] proposed that one state of the organized epicuticular free lipid layer exists as a “crystalline wax surface bloom” over pore canals. Our heating data showed that the shapes of the domes in domed pores and of nipples in nipple-pores did not change when heated to 50 °C. The behavior of these domes and nipples under high heat would seem to be consistent with a crystalline wax type of composition.

We do not know whether the exudates residing within pores (nipples) or protruding from the pores (domes) impede, facilitate or do nothing at all to affect the pore-uptake of pheromone molecules. It does seem possible that even in “open” pores there could be very small amounts of this same exudate deep with the pores that we could not detect with the AFM probe. Thus, an exudate substance of any of these three sizes might somehow be assisting in the passage of odorants down the pores, even though when they are larger, as with the nipples and domes, they appear to be hindering passage down a pore to varying degrees.

Our study also showed that there are variations across species with regard to the topographies, spacings and densities of pores and ridges arranged on trichoid sensilla. A previous recent AFM study [17] focused on aspects of how ridges and pores may be positioned optimally to create favorable turbulence that promotes a favorable aerial transport and deposition of *B. mori* pheromone molecules onto the pores. The measurements in that study, and those of Louden and Koehl [31], were calculated using sensilla of male moths with bipectinate (feathery) antennae. In our study, most of the male trichoid sensilla were from filiform (long, threadlike) antennae, except for those of *L. dispar* and *M. sexta*. We found that the distances of pores from ridges varied among the species, even though the ridge heights seemed quite similar. Louden and Koehl [31] concluded that the fluid mechanics governing molecular activities around sensilla would be influenced more by diffusion than by fluid flow. The differences across the species used in our study in their pore-to-ridge spacings, pore densities and inter-pore spacings therefore might be more related to differences in diffusion coefficients and polarities of the different types of pheromone molecules of each species and any species-specific differences in lipid coatings. If pheromone molecules develop a static electric charge as they are released from female glands, or if males pick up charge during take-off and flight, these could also influence the ability of pheromone molecules to be captured by trichoid sensillar surfaces and interact with the lipids in the epicuticle to be transported quickly down through the pores.

## Data Availability

Data is available from lead author T.C.B. upon request.

## Supplementary Materials

The following are available online at https://www.mdpi.com/article/10.3390/insects13050423/s1, Figure S1, Pore and Pore-Ridge Spacings in nm; Figure S2, Nipple-Pore and Open Pore Pore Diameters; Figure S3, Nipple Pore and Open Pore Pore Depths; Figure S4, Nipple and Dome Heights; Figure S5, Nipple and Dome Diameters.
