# Supplementary material for: Surface Properties and Architectures of Male Moth Trichoid Sensilla Investigated Using Atomic Force Microscopy
Source: Insects. 2022 Apr 30;13(5):423. doi: 10.3390/insects13050423 (PMC9146897; doi:10.3390/insects13050423)
Supplement: Supplementary file 1 [file insects-13-00423-s001.zip › insects-1666163-supplementary.pdf]

Supplemental Figures

Figure S1

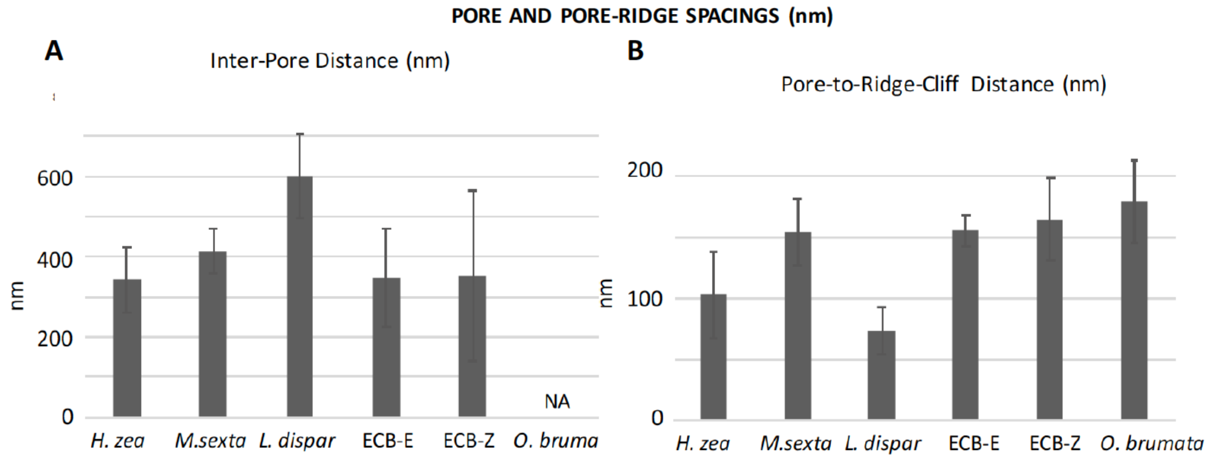

Figure S1. Distances, in nm, of spacings between nearest-neighbor pores and also spacings between a pore and its nearest proximal ridge-cliff. The labels, "ECB-E" and "ECB-Z", refer to the *E*- and *Z*-strains, respectively, of *Ostrinia nubilalis* (European corn borer). Error bars denote standard deviations. **A**) Mean nearest-neighbor inter-pore distances, in nm, of the five species' trichoid sensilla. There were only single pores visible on the sensilla of *Operophtera brumata*, and therefore no inter-pore distances could be measured. N = 15, 10, 17, 8, and 7 inter-pore distances measured for *Helicoverpa zea*, *Manduca sexta*, *Lymantria dispar*, *Ostrinia nubilalis* E-strain ("ECB-E"), and *O. nubilalis* Z-strain ("ECB-Z"), respectively. **B**) Mean distances of pores to the base of their nearest proximal ridge-cliff. The pores of *L. dispar* and *H. zea* were positioned closer to the bases of ridges than were the pores of other species. N = 14, 10, 16, 7, 9, and 9 pore-to-ridge distances measured for *H. zea*, *M. sexta*, *L. dispar*, *O. nubilalis* E-strain, *O. nubilalis* Z-strain, and *O. brumata*, respectively.

Figure S2

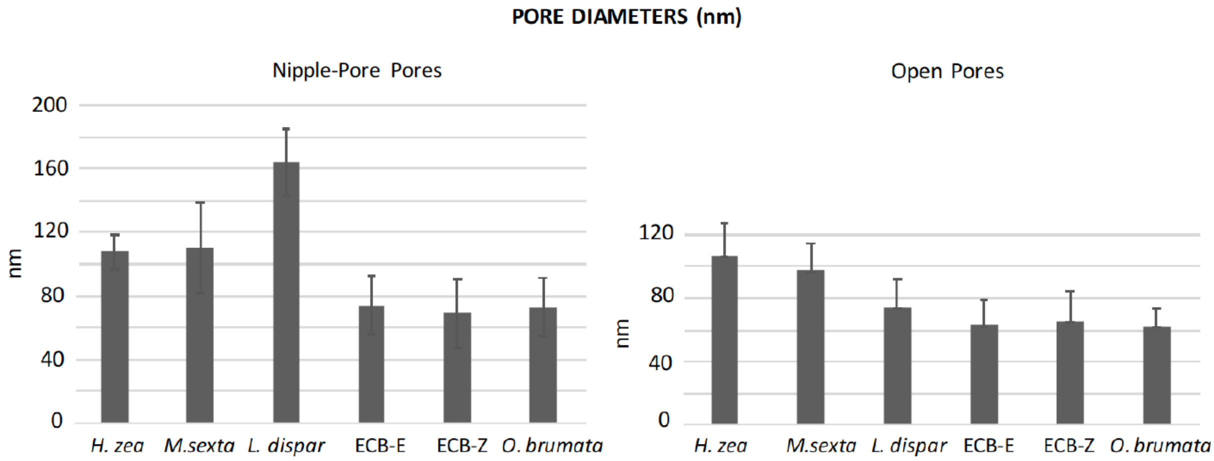

Figure S2. Mean diameters, in nm, of the nipple-pores and open pores of the five species used in this study. Please refer to Figure 2 for measurement protocols. The labels, "ECB-E" and "ECB-Z", refer to the *E*- and *Z*-strains, respectively of *Ostrinia nubilalis* (European corn borer). Error bars denote standard deviations. The diameters of the pores of domed pores could not be measured due to the domes covering the outer rims of the pores.

A) Mean diameters of nipple-pore pores. N = 18, 26, 12, 19, 5, and 2 nipple-pore pore diameters measured for *Helicoverpa zea*, *Manduca sexta*, *Lymantria dispar*, *Ostrinia nubilalis* E-strain, *O. nubilalis* Z-strain, and

Operophtera brumata, respectively. **B**) Mean diameters of open pores. N = 11, 5, 18, 8, 25, and 16 open pore diameters measured for *H. zea*, *M. sexta*, *L. dispar*, *O. nubilalis* E-strain, *O. nubilalis* Z-strain, and *O. brumata*, respectively.

Figure S3

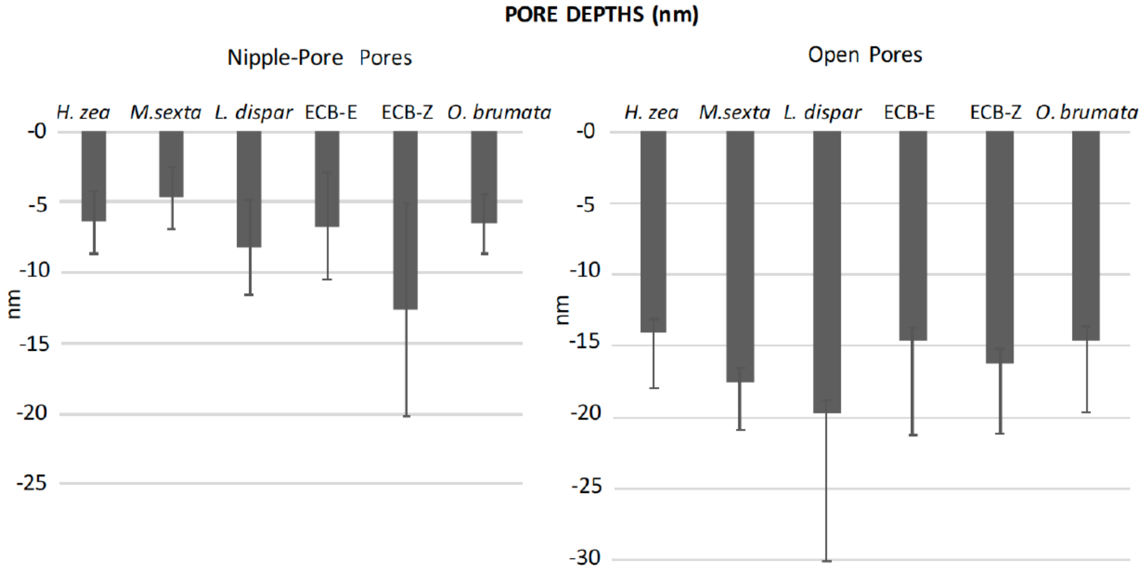

Figure S3. Mean depths of nipple-pore pores and open pores measured for the five species. Please refer to Figure 2 for measurement protocols. The labels, "ECB-E" and "ECB-Z", refer to the *E*- and *Z*-strains, respectively, of *Ostrinia nubilalis* (European corn borer). Error bars denote standard deviations. **A**) Mean depths of nipple-pore pores. N = 18, 26, 12, 19, 5, and 2 nipple-pore pore depths measured for *Helicoverpa zea*, *Manduca sexta*, *Lymantria dispar*, *Ostrinia nubilalis* E-strain, *O. nubilalis* Z-strain, and *Operophtera brumata*, respectively. **B**) Mean depths of open pores. N = 11, 10, 18, 8, 25, and 16 open pore diameters measured for *H. zea*, *M. sexta*, *L. dispar*, *O. nubilalis* E-strain, *O. nubilalis* Z-strain, and *O. brumata*, respectively.

Figure S4

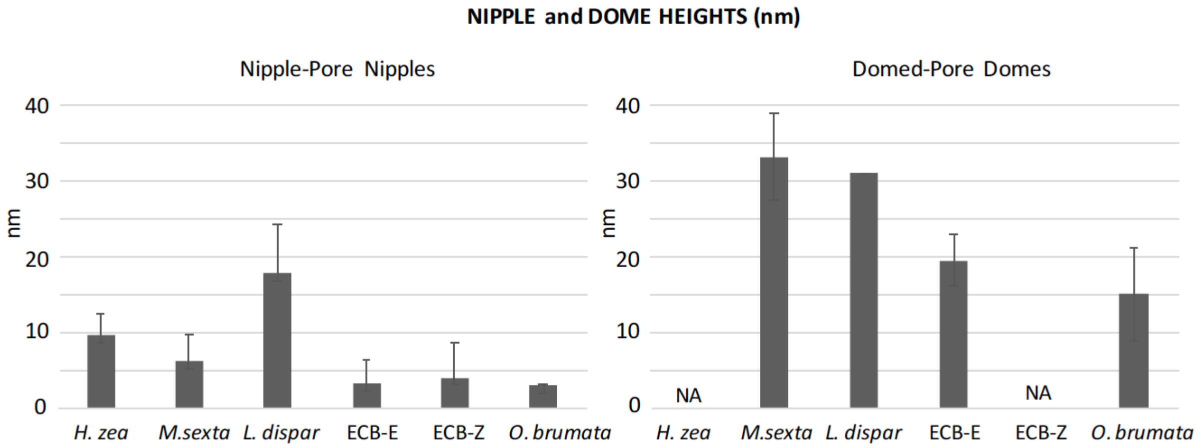

Figure S4. Mean heights of nipple-pore nipples and domed-pore domes measured for the five species. The labels, "ECB-E" and "ECB-Z", refer to the *E*- and *Z*-strains, respectively, of *Ostrinia nubilalis* (European corn borer). Error bars denote standard deviations. **A**) Mean heights of nipple-pore nipples. N = 18, 26, 12, 19, 5, and 2 nipple-pore nipple heights measured for *Helicoverpa zea*, *Manduca sexta*, *Lymantria dispar*, *Ostrinia nubilalis* E-strain, *O.*

nubilalis Z-strain, and *Operophtera brumata*, respectively. **B**) Mean heights of domed-pore domes. N = 4, 1, 4, and 4 dome heights measured for *M. sexta*, *L. dispar*, *O. nubilalis* E-strain, and *O. brumata*, respectively. No domed pore domes were found for *H. zea* and *O. nubilalis* Z-strain in our AFM scans.

Figure S5

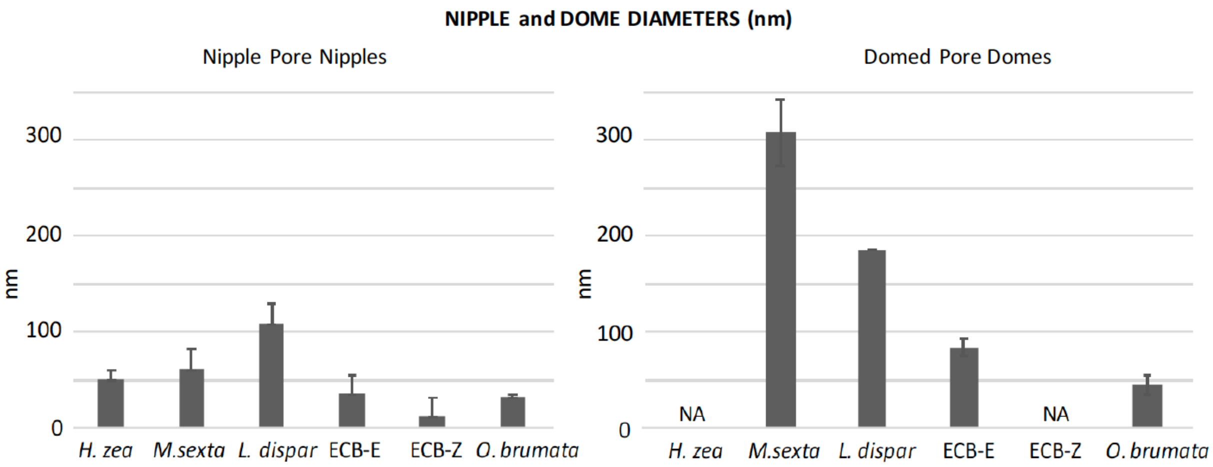

Figure S5. Mean diameters of nipple-pore nipples and domed pore domes measured for the five species. The labels, "ECB-E" and "ECB-Z", refer to the *E*- and *Z*-strains of *O. nubilalis* (European corn borer) respectively. Error bars denote standard deviations. **A**) Mean diameters of nipple-pore nipples. Please refer to Figure 2 for measurement protocols. N = 18, 26, 12, 19, 5, and 2 nipple-pore nipple diameters measured for *Helicoverpa zea*, *Manduca sexta*, *Lymantria dispar*, *Ostrinia nubilalis* E-strain, *O. nubilalis* Z-strain, and *Operophtera brumata*, respectively. **B**) Mean diameters of domed pore domes. N = 4, 1, 4, and 4 dome diameters measured for *M. sexta*, *L. dispar*, *O. nubilalis* E-strain, and *O. brumata*, respectively. No domed pore domes were found for *H. zea* and *O. nubilalis* Z-strain in our AFM scans.
